# Do Staple Food Consumption Patterns Affect Household Food Waste in Rural China?

**DOI:** 10.3390/foods14091584

**Published:** 2025-04-30

**Authors:** Nanyan Hu, Jiachen Guan, Yonghao Hu, Laping Wu

**Affiliations:** 1 College of Economics and Management, China Agricultural University, Beijing 100083, China; hny@cau.edu.cn (N.H.); cem-hyh@cau.edu.cn (Y.H.); wulp@cau.edu.cn (L.W.); 2 Institute of Technology and Economy of Grain Industry, Academy of National Food and Strategic Reserves Administration, Beijing 100045, China

**Keywords:** staple food consumption pattern, household, food waste, Tobit model

## Abstract

This study utilizes a national database derived from large-scale surveys to examine the impact of staple food consumption patterns on household food waste in rural China. Using the Tobit model, the findings show that staple food consumption significantly influences food waste. Specifically, southern households, where rice is the staple food, waste 44% more food than their northern counterparts, where wheat is the staple. To address potential self-selection bias, the study employs the propensity score matching (PSM) model to validate the robustness of these results. Further heterogeneity analysis shows that the effect of staple food consumption on food waste is more pronounced in larger rural households with children. These results underscore the importance of dietary culture in explaining regional disparities in food waste across China.

## 1. Introduction

In recent years, global food insecurity has been exacerbated by the combined impacts of the pandemic, climate change, and regional conflicts among major food-producing nations (Clapp and Moseley, 2020) [1]. According to the 2023 World Food Programme report, approximately 258 million people in 58 countries faced severe food insecurity in 2022. The Food and Agriculture Organization (FAO) reports that one-third of food produced for human consumption is wasted, amounting to roughly 1.3 billion tons annually, valued at over USD 750 billion (FAO, 2020) [2]. Reducing food waste is critical to improving food production, trade, and distribution to address food insecurity.

As one of the world’s largest food producers and consumers, China’s food security is globally significant. Research on food waste in China offers valuable insights for other countries confronting similar challenges. In 2023, China’s food production exceeded 650 million tons for the eighth consecutive year, with per capita food output reaching 483 kg and cereal self-sufficiency remaining above 95% (Wu, 2021) [3]. However, approximately 35 million tons of food are wasted annually in China, accounting for nearly 6% of its total food production, enough to feed more than 200 million people (IFAD, 2020) [4]. According to the 2023 China Food and Nutrition Development Report, the overall food loss and waste rate in China is approximately 22.7%, with a total loss of 460 million tons in 2022 (Food and Nutrition Development Institute, 2023) [5]. Thus, reducing food waste is essential for ensuring China’s long-term food security (China Economic Net, 2016) [6].

Households are central to food consumption and are widely recognized as significant contributors to food waste (Lin and Guan, 2021) [7]. For example, in the United Kingdom, household food waste accounts for a substantial 70% of the total waste (WRAP, 2023) [8]. In developing countries like India, households contribute approximately 50% of food waste (Thi et al., 2015) [9]. Similarly, food waste constitutes 30% of the overall waste stream in Iran, and in the United States, households are responsible for 35% of total food waste. According to the UNEP (2021) [10], households accounted for 61% of global food waste in 2019.

Extensive research has investigated the determinants of household food waste at the micro level, encompassing demographic characteristics (Wu et al., 2019; Chalak et al., 2019) [11,12], economic decision-making (Segre et al., 2014) [13], nutrition literacy (Min et al., 2021) [14], and technological interventions (Schuster et al., 2022) [15]. However, these studies frequently neglect the pivotal behavioral and psychological dimensions that underpin wasteful practices. For example, habitual over-purchasing may arise from deeply ingrained consumer routines (Sheth, 1981) [16], while reluctance to discard edible food is often driven by psychological factors such as guilt or aversion to waste (Graham-Rowe et al., 2014) [17]. Additionally, cognitive biases, such as the tendency to overestimate food needs, exacerbate wasteful behaviors (Tversky and Kahneman, 1974) [18]. Despite these insights, the macro-cultural perspective, particularly how regional variations in staple food consumption patterns shape household waste, remains underexplored (Secondi et al., 2015) [19]. The dietary cultural divide between northern and southern China offers a compelling lens for examining this issue. Talhelm (2020) [20] demonstrates in a cross-cultural study that residents of rice-based and wheat-based regions exhibit marked differences in cognition and social behavior, potentially influencing resource utilization efficiency through distinct consumption habits. Although Qian et al. (2022) [21] report that southern students, predominantly rice consumers, generate significantly more food waste than their northern counterparts, their findings are constrained by a homogeneous sample (87% aged 18–22) and a specific context (canteen dining), limiting generalizability (Stancu et al., 2016) [22]. To address these gaps, this study leverages cross-regional household data and employs the Tobit model to account for confounders such as household size and storage facilities (Koivupuro et al., 2012) [23], examining the nonlinear effects of staple food consumption culture on household food waste. This approach not only builds on the established frameworks, but also broadens the analytical scope by incorporating a cultural geography perspective, informed by cross-cultural consumption research (Zhang et al., 2020) [24]. Current scholarship, however, reveals two key methodological shortcomings: first, many studies rely on linear models to analyze food waste data with truncation characteristics (Stancu et al., 2016) [22], overlooking the risk of bias in parameter estimation due to zero-inflated distributions; second, regional dietary heterogeneity is often relegated to a control variable rather than treated as a central mechanism (Schanes et al., 2018) [25].

To address the first issue, this study employs the Tobit model, which is supported by dual theoretical foundations: food waste behavior aligns with the “latent variable decision mechanism” in the theory of planned behavior (Ajzen, 1991) [26], and the validity of the model in household consumption research has been validated (Zhang et al., 2020) [24]. To address the second issue, we constructed an analytical framework based on the differences in dietary structures between northern and southern China: Qian et al. (2022) [21] found a significant correlation between staple food choices (rice versus wheat) and food waste, but their study suffered from a population coverage bias. Liu et al. (2021) [27], in the national nutrition survey, confirmed that rural households exhibit strong cultural inertia and geographical stickiness in their staple food consumption patterns, which provides the theoretical basis for focusing on rural samples in this study. This study uses survey data from 1632 rural households in China to explore the impact of staple food consumption patterns on food waste. We recorded food waste in these households over three consecutive days to ensure accuracy. By examining the role of dietary culture, this research contributes to a deeper understanding of food waste, which is essential for developing effective waste-reduction strategies. Our findings align with the United Nations Sustainable Development Goals by identifying the key factors contributing to food waste.

This study offers three primary contributions to the literature on household food waste. First, it elucidates the cultural transmission mechanisms underlying staple food consumption patterns. Prior research has often reduced food culture to a simplistic geographic proxy, neglecting the role of staple food preferences as carriers of cultural identity. Through rigorous empirical analysis, this study examines how regional dietary traditions shape food waste behaviors via staple food choices, thereby addressing an empirical gap in Talhelm’s (2020) [20] Cultural Cognition Theory within the context of household consumption. Second, it tackles the truncated nature of food waste data by integrating the Tobit model with marginal effect decomposition (Wooldridge, 2010) [28], effectively mitigating the estimation biases inherent in traditional linear models when applied to zero-inflated distributions (Stancu et al., 2016) [22]. Third, the study disentangles the nuanced relationship between cultural differences and specific types of food waste. While existing scholarship predominantly focuses on aggregate food waste volumes (Liu et al., 2021) [27], it largely overlooks how cultural attributes differentially affect waste across food categories. Notably, this analysis reveals that in rice-cultivating regions, inadequate storage practices and limited cooking expertise lead to disproportionately higher flour waste. These findings provide a culturally informed perspective that enhances the framework of the United Nations Environment Programme’s “Precision Waste Reduction Policy”.

The remainder of the paper is organized as follows: Section 2 introduces our data and sources. Section 3 presents our main empirical results and various robustness checks. Section 4 presents a discussion. Section 5 concludes the paper.

## 2. Theoretical Framework and Hypothesis

The historical divergence in staple crop cultivation between northern and southern China has profoundly shaped socio-cultural norms and food waste behaviors. For millennia, rice-based agriculture in the South demanded intensive water management and labor coordination, in sharp contrast to the wheat-centric practices of the north. Unlike wheat cultivation, which largely depends on natural rainfall, rice production necessitated intricate irrigation systems that often extended across multiple households. The public goods nature of these systems fostered interdependencies, as individual decisions, such as excessive water extraction, generated externalities that affected neighboring farmers, requiring village-level coordination to construct and maintain irrigation canals (Talhelm et al., 2014) [29]. Furthermore, rice farming entailed substantially greater labor demands than wheat farming, with activities like seedling transplantation and harvesting necessitating synchronized labor exchanges among families to align with tight seasonal deadlines (Bray, 2023) [30]. These collective agricultural practices entrenched norms of reciprocity and prioritized group cohesion over individual preferences, leaving a lasting imprint on regional food-related behaviors.

In contrast, northern China’s wheat-based agriculture was characterized by fewer coordination demands. Wheat cultivation was less reliant on irrigation and more dependent on natural rainfall, reducing the need for coordinated water management. The labor demands of wheat farming were also smaller and more flexible in terms of timing, allowing households to operate independently without relying on communal labor pools (Elvin, 1973) [31]. This autonomy fostered individualistic pragmatism, with greater emphasis placed on the needs of the nuclear family over those of the broader community.

Although urbanization has reduced direct engagement with agriculture, the values rooted in these agricultural practices continue to influence food behaviors through intergenerational transmission. In rice-growing regions, collectivist values, such as prioritizing group harmony and social reputation, continue to shape food consumption patterns. These traditions lead to over-procurement, as southern households historically stocked rice to fulfill communal obligations, such as hosting guests or preparing for ceremonial feasts. Despite improved market accessibility, this habit of over-purchasing persists in modern-day practices, often reinforced by behavioral inertia and entrenched consumer routines (Sheth, 1981) [16]. Moreover, psychological factors, such as the fear of scarcity or guilt associated with failing to meet social expectations, further perpetuate this tendency (Graham-Rowe et al., 2014) [17]. Additionally, the collective dining culture in southern regions demands multiple dishes per meal, which increases ingredient variety and often results in surplus food that is not fully utilized, leading to waste. This behavior is compounded by cognitive biases, such as overestimating food needs to ensure abundance, a pattern linked to decision-making under uncertainty (Tversky and Kahneman, 1974) [18]. These behavioral and psychological dimensions highlight how cultural legacies interact with individual perceptions to drive food waste in contemporary settings.

In contrast, the self-reliant culture of northern wheat-consuming households fosters pragmatic food management practices. Meals in these regions typically feature fewer staple-based dishes, which limits ingredient variety and reduces the likelihood of spoilage. Moreover, northern households often adopt a just-in-time procurement strategy, opting for frequent, smaller purchases tailored to immediate needs rather than the social stockpiling prevalent in the south.

By synthesizing these agricultural and cultural mechanisms, we propose the following hypothesis:

**H1:**  Households in southern China (rice-dominant) generate higher food waste than those in northern China (wheat-dominant), mediated by over-procurement and collectivism-driven and diversified meal preparation practices.

## 3. Materials and Methods

### 3.1. Sample Collection

In 2016, we collaborated with the Office of Rural Fixed Observation Points under the Ministry of Agriculture and Rural Affairs to assess the extent of grain loss and waste in China. The Ministry had established a comprehensive rural survey system designed for the ongoing monitoring of smallholder farms across all 31 provinces. To support this investigation, we designed a survey questionnaire and, with assistance from the system’s staff, conducted field surveys. The survey included 1632 households spanning 21 provinces, autonomous regions, and municipalities. Participants were thoroughly informed of the study’s objectives and retained the right to withdraw at any time without providing justification. Ethical approval was secured from the Institutional Review Board (IRB) of China Agricultural University, and all research procedures complied with the ethical standards set forth in the Declaration of Helsinki.

A multistage random sampling procedure was implemented to secure a representative and comprehensive sample. Initially, the study accounted for geographical location, economic development levels, and consumer patterns, which differ markedly between northern and southern China. Accordingly, regions were selected to reflect this North–South divide: the North encompasses northern, northeastern, and northwestern China, while the South includes southeastern, central southern, and southwestern regions.

Subsequently, a comprehensive list of villages was obtained from county statistical bureaus or relevant authorities. To ensure sample representativeness, villages were stratified by economic development level and geographical location. Within each stratum, one village was randomly selected using a random number generator or lottery method. Following village selection, their baseline characteristics—such as population size and economic status—were verified to confirm alignment with the study’s objectives. Finally, rural households within each selected village were randomly chosen, and their food waste behaviors were observed over three consecutive days.

The survey collected data across three primary domains: (i) rural household characteristics, (ii) cooking practices, and (iii) household food waste. Samples with incomplete food waste data or waste rates falling outside the 0% to 60% range were excluded from analysis. After applying these criteria, the final dataset comprised 1632 rural households.

### 3.2. Food Waste Measurement

The Food and Agriculture Organization (FAO) has updated its estimations, indicating 14% food loss (occurring between farm and retail levels) and 17% food waste (resulting from households and food services) (FAO, 2019) [32]. This study defines food waste as waste generated within households only.

Household food waste is typically categorized into five stages: (1) food purchasing, (2) storing, (3) preparing, (4) consuming, and (5) handling (Buzby and Hyman, 2012; Parfitt et al., 2010; WRAP, 2007) [33,34,35]. This study focuses on the consumption stage, which is defined as the food that remains uneaten but is still edible after a meal. This definition aligns with previous research and follows the guidelines for the Waste and Resources Action Program (WRAP).

The accurate measurement of food waste is essential for reliable data. Various methods have been used in the literature, including food waste diaries, photographic evidence, qualitative interviews, and self-reports. These methods range from keeping food waste diaries and using structured self-report forms to self-collecting data and conducting observational assessments with photographs. Each approach has its advantages and limitations. Recent studies have predominantly relied on the kitchen diary method and self-reported food waste quantities, which are also adopted in this study to ensure consistency with the established practices.

In this survey, households were the unit of analysis, and food waste was measured over three consecutive days. Dining out was excluded from the study. The survey focused on the variety of foods consumed for breakfast, lunch, and dinner. The food waste ratio was calculated by comparing the weight of discarded edible food to the total weight of the prepared meal. The food waste was recorded as zero if a family skipped a meal. Any uneaten food, even if fed to pets, was considered waste. In addition to staple food waste, other food waste was assessed through visual inspection based on the specific situation. The designated respondent was the household bookkeeper, familiar with food waste patterns.

The survey collected data on nine food categories, reflecting rural China’s typical dietary consumption patterns. These categories included: (1) staple food noodles; (2) staple food rice products; (3) potatoes; (4) soybean products; (5) pork; (6) other meats; (7) poultry; (8) aquatic products; and (9) eggs.

Food waste was measured as the proportion of discarded food, calculated by quantifying the weight of the discarded food (Qian et al., 2021) [36]. The following is the calculation formula:

(1) Wi=wfipfi

where *W_i_* is the food waste reported among rural households, *wf_i_* is the weight of food discarded by rural households that is still considered edible, and *pf_i_* is the total weight of food utilized for cooking purposes. When rural households did not undergo meal inspections or dined away from home, it was recorded as “-”. In addressing food waste calculation in multi-member households, this study adopts a household-level aggregation approach. Specifically, food waste is recorded at the household level, regardless of the number of household members involved in meal preparation, rather than being adjusted per capita. This approach is justified by several factors: First, food waste is closely related to the household’s overall consumption patterns, such as purchasing decisions, storage practices, and cooking methods—rather than individual behaviors. For example, waste resulting from collective purchasing decisions or improper storage cannot be easily attributed to specific members of the household. Second, requiring individual members to report their own waste would increase the complexity of data collection and could lead to inaccuracies due to recall bias or social desirability bias, such as underreporting waste. Lastly, household-level aggregation better aligns with the practical needs of policy interventions, such as educational programs or subsidies targeting households. Therefore, adopting this approach ensures consistency and enhances the applicability of the food waste calculation in the study.

### 3.3. Staple Food Consumption Pattern

In this study, staple food consumption patterns serve as the independent variable. Prior research has established a robust association between dietary structure and household food waste behaviors (Phasha et al., 2020) [37]. Given China’s vast geographical diversity, food consumption patterns exhibit significant regional variation. In northern China, wheat predominates as the staple food, while rice prevails in the southern regions (Qian et al., 2021) [36]. This distinction mirrors agricultural practices, with wheat cultivation concentrated in the North and rice production in the South.

To address additional regional disparities, we incorporated food consumption patterns from eastern and western China. A review of the existing literature identified key wheat-producing provinces, including Henan, Shaanxi, and Shandong in the East, and Gansu, Xinjiang, and Tibet in the West. The dataset delineates the origins of wheat in these regions, as presented in Table 1. Accordingly, households in these wheat-producing areas were categorized as wheat-based (northern) households, assigned a value of 0, while those in predominantly rice-growing regions were designated as rice-based (southern) households, assigned a value of 1.

### 3.4. Control Variables

Building on prior research into food waste in rural households, this study incorporates three control variables: individual-level characteristics (Ilakovac et al., 2020) [50], cooking methods, and family-level characteristics (Filipova et al., 2017) [51].

The average food waste ratio in the sample is 2.7%, with a standard deviation of 1.619, reflecting moderate variability across households. In terms of staple food consumption patterns, 66.1% of households predominantly consume rice, while the remainder rely on wheat. The average age of household heads is 57 years, and their mean educational attainment is 7.616 years, suggesting that they are typically older with a moderate level of schooling. Regarding cooking technology, 69.1% of households utilize modern cooking appliances, indicating widespread adoption of contemporary equipment. In food preparation preferences, 76.7% favor stir-fry techniques, underscoring a prevalent culinary practice. Gender dynamics in cooking reveal that only 8.6% of households have male primary meal preparers, highlighting the predominance of female-led meal preparation. Cooking habits further show that 72.7% of households prepare dishes for a single meal, pointing to a common pattern of meal planning. Dining out is reported by 50% of households, reflecting its commonality, while 21.6% use electricity as their primary cooking fuel, with the majority still depending on traditional fuel sources. Condiment use is nearly ubiquitous, with 89.2% of households regularly incorporating seasonings into their cooking. In terms of food disposal, only 3.4% discard unprocessed food, suggesting a general inclination towards minimizing waste.

Family characteristics include household size, average body mass index (BMI), household income, and the distance to primary stores. Prior research indicates that larger households tend to produce more food waste, likely due to the need to purchase diverse food items to accommodate varied preferences (Dobernig and Schanes, 2019) [52]. Dobernig and Schanes further identify average BMI and household income as critical factors influencing food waste levels in rural settings. Definitions and descriptive statistics for all variables are provided in Table 2.

### 3.5. Identification Strategy

To investigate the impact of staple food consumption patterns on household food waste in rural China, this study introduces the following basic econometric model:

(2) $$FW_{i}=\beta_{0}+\beta_{1}{Crop}_{i}+\beta_{2}X_{i}+\varepsilon_{i}$$

where *FW_i_* represents the food waste rate of rural household *i*, ranging from 0 to 1. *Crop_i_* indicates the staple food consumption pattern, distinguishing between southern and northern households. *X_i_* denotes a set of control variables influencing food waste in rural households. $\beta_{0}$ is a constant term. The coefficients $\beta_{1}\sim \beta_{2}$ are to be estimated. $\varepsilon_{i}$ represents the error term.

Given that the dependent variable exhibits a typical truncated distribution, ranging from 0 to 1 with 28.6% of observations at zero, the traditional ordinary least squares (OLS) method may yield biased estimates (Maddala, 1983) [53]. Consequently, this study employs the Tobit model based on two primary considerations.

First, following the criterion for handling restricted dependent variables proposed by Wooldridge (2010) [28], the Tobit model is particularly suitable when the dependent variable displays a pronounced clustering effect at a threshold value. Through its latent variable framework, the Tobit model provides a more accurate depiction of the data-generating process. Specifically, the latent propensity for food waste is defined as *Y*^∗^, with the observed value *Y* expressed as:

(3) $$Y=X\beta +\varepsilon ,\;\varepsilon |X\sim Normal(0,\sigma^{2})$$

This latent variable framework aligns closely with the characteristics of the food waste rate in this study, which exhibits both left truncation (zero waste) and right truncation (complete waste). Wooldridge notes that this approach outperforms traditional linear regression methods when addressing truncated data with boundary accumulation (Wooldridge, 2010, p. 567) [28]. Furthermore, Greene emphasizes that the Tobit model is well suited to dependent variables constrained within a specific range and featuring a substantial proportion of boundary values, a condition met by the food waste rate distribution in this study (Greene, 2012, p. 827) [54].

Second, to assess the appropriateness of the Tobit model, this study employs the Vuong test (Vuong, 1989) [55] to compare its goodness-of-fit against that of the OLS and Two-Part models. The results indicate that the Tobit model significantly outperforms the alternatives (Vuong likelihood ratio statistically significant at *p* < 0.05), demonstrating a superior fit for the data in this study. While the Two-Part model can be effective in certain contexts for separately modeling the mechanisms generating zero and continuous values (Cragg, 1971) [56], the Vuong test results suggest that it does not outperform the Tobit model in this case. Additionally, Amemiya argues that the Tobit model offers theoretical robustness when handling truncated data generated by a single latent process, further justifying its adoption here (Amemiya, 1984, p. 12) [57].

### 3.6. Food Waste in Different Regions Among Rural Households

Given China’s vast geographical diversity, regional variations in dietary patterns are pronounced. This study first provides an overview of food waste rates across the entire sample, then compares waste proportions between southern rice-based and northern wheat-based regions. Table 3 details the waste proportions across nine food categories.

On average, rural households in China discard 2.70% of their food. Among the food types examined, rice, potatoes, and flour products exhibit the highest waste rates, while beef and pork demonstrate the lowest. This pattern is largely attributable to food prices: staple foods such as rice and potatoes are relatively inexpensive, encouraging less caution in their use, whereas meat products like beef and pork, being more costly, are managed more judiciously, resulting in reduced waste.

Regionally, rice-based households exhibit an average food waste rate of 2.97%, modestly exceeding the 2.32% observed in wheat-based households. In rice-based regions, waste rates across various food categories, including rice, potatoes, soybean products, pork, aquatic products, and eggs, are 4.50%, 3.10%, 2.06%, 1.49%, 2.34%, and 1.39%, respectively. These figures are notably higher compared to waste rates in wheat-based regions. Conversely, in wheat-growing regions, waste rates for specific categories such as flour, beef, and poultry surpass those in rice-based regions, highlighting distinct regional patterns.

This comparative analysis of food waste proportions across regions provides a clearer picture of the prevailing food waste landscape in China. These insights offer policymakers a robust foundation for designing targeted, region-specific strategies to reduce food waste effectively.

## 4. Results

### 4.1. Baseline Results

Based on the empirical findings from the Tobit model, specifically the North–South divide, this study examines the impact of staple food consumption patterns on household food waste. The results show a significant positive correlation between staple food consumption patterns and household food waste. On average, rice-based households in the southern regions waste about 0.440 more food than wheat-based households in the North.

Why do rice-based households produce more food waste than their wheat-based counterparts? Rice cultivation in the South is labor-intensive, requiring substantial effort for planting and maintenance. Additionally, the rice field irrigation system relies on neighbors’ collective efforts. This cultivation model fosters a strong sense of cooperation within families and among neighbors, contributing to a collectivist culture. In contrast, wheat cultivation in the North is more extensive and relies on natural precipitation or individual irrigation wells, creating a more independent, individualistic culture.

Rice-producing villages have developed strong norms of reciprocity to meet the labor demands, which are double those of dryland crops like wheat (Talhelm, 2014) [29]. As a result, in rice-based rural households, sharing meals with others is common, optimizing collective utility. However, shared eating habits often lead to higher food waste, as there is less individual accountability for portions. This behavior is exacerbated by psychological tendencies, such as the reluctance to discard surplus food due to guilt or social pressure to uphold communal norms (Graham-Rowe et al., 2014) [17]. Moreover, the habitual preparation of excess food, rooted in cultural expectations of abundance, reflects behavioral inertia that resists change even in modern contexts (Sheth, 1981) [16]. In contrast, wheat-based rural households, with more independent eating habits driven by personal choice, tend to waste less food. This difference is further influenced by cognitive biases, such as the overestimation of food needs in collectivist settings to ensure sufficiency for all, a pattern tied to decision-making under uncertainty (Tversky and Kahneman, 1974) [18]. These findings underscore how behavioral and psychological factors, intertwined with cultural practices, contribute to the observed disparities in food waste.

As for other variables, the education level of the household head significantly reduces food waste. Those with a higher level of education tend to exhibit more vigorous saving habits. Additionally, modern cooking tools reduce food waste more effectively than traditional ones. Modern cooking containers have standardized measurements, allowing for more accurate food preparation. Planned cooking habits also show a strong negative association with food waste, statistically significant at the 5% confidence level, providing empirical support for the extended theory of planned behavior. By comparing the food waste behaviors of households using modern cooking tools with those using traditional tools, the study found that households using modern tools waste less food. This result may be attributed to the precise control features of modern tools, such as the timer function of rice cookers and the rapid heating capability of microwaves, which help reduce waste caused by the improper cooking or over-preparation of food. However, the impact of using modern tools on household food waste was not statistically significant, potentially influenced by factors such as cooking skills and the compatibility of the tools’ features.

Furthermore, household size is positively associated with food waste. Larger households tend to prepare more food, which results in more waste. This finding aligns with Fami’s research (2019) [58]. Lastly, while a negative relationship between distance to primary stores and food waste was observed, this result was not statistically significant.

After controlling for other factors, the “cook gender” and “cooking habits” variables also show some impact on household food waste, with the negative correlation for cook gender indicating that men may be more inclined to reduce food waste. Household size and the number of children also show some relationships with food waste. Overall, the results in Table 4 suggest that staple food consumption patterns play a significant role in household food waste, while other individual and household factors, such as age, education, and household size, also influence household food waste.

This study further investigates the influence of staple food consumption patterns on various types of food waste, with the findings detailed in Table 5. Relative to wheat-based households, rice-based households demonstrate significantly greater food waste, particularly for wheat products, rice, potatoes, and poultry. In contrast, no statistically significant effects were observed for seafood, pork, lamb, or egg products.

Drawing on the data in Table 5, this analysis assesses the relationship between staple food consumption patterns and household food waste across multiple food categories. The table reports coefficients for each food type, with aggregate values reflecting the overall association between consumption patterns and waste, bolstered by the inclusion of control variables to ensure robustness. The results reveal significant correlations for several staple foods: rice, flour, and potatoes exhibit positive and statistically significant relationships with household food waste, with coefficients of 0.219, 0.185, and 0.108, respectively, all significant at the 1% level.

These findings suggest that higher consumption of these staple foods is associated with greater food waste, potentially due to factors such as over-purchasing or improper storage practices. This tendency may be amplified by behavioral patterns, such as habitual over-preparation rooted in routines of ensuring abundance (Sheth, 1981) [16], and psychological factors, including the reluctance to discard food due to guilt or perceived waste aversion (Graham-Rowe et al., 2014) [17]. In contrast, soy consumption is negatively associated with food waste (−0.067), significant at the 10% level, indicating that higher soy consumption may reduce food waste, possibly due to more efficient utilization or lower spoilage rates of these items. It is suggested that the consumption patterns for other food items, such as pork, beef, and aquatic products, do not significantly impact household food waste. This could reflect greater caution in handling meat products, driven by higher perceived value or cost, which mitigates waste through more deliberate portioning (Quested et al., 2013) [59]. However, poultry consumption demonstrates a positive and statistically significant relationship with food waste (0.093), implying that increased poultry consumption is linked to higher waste, which could be attributed to issues such as improper portioning or preparation practices, compounded by cognitive biases like overestimating portion needs (Tversky and Kahneman, 1974) [18]. These results underscore the importance of targeted interventions tailored to specific food types to mitigate household food waste.

### 4.2. Robustness Test I

Given the potential heterogeneity in factors such as economic status and food preferences across rural households, estimation errors may arise. To address this, this study adopts the propensity score matching (PSM) method, developed by Rosenbaum and Rubin (1983) [60], to mitigate selection bias. Table 6 presents the results derived from nearest neighbor matching, radius matching, and kernel matching techniques. These findings align closely with the baseline regression results, reinforcing the robustness of the study’s conclusions.

### 4.3. Robustness Test II

Building on previous research, this study introduces a revised independent variable using the Qingling Mountains and Huaihe River as a boundary to categorize rural households (Qian et al., 2021) [36]. Rural households south of this boundary are assigned a value of 1, while those located to the north are assigned a value of 0.

As shown in Table 7, rural households in rice-based consumption areas are more likely to waste food than those in wheat-based regions. These results are consistent with the baseline regression findings, and robustness tests confirm the reliability of these conclusions.

### 4.4. Heterogeneity Analysis

The primary source of heterogeneity in this study is household income, a key determinant of food waste. Rural households were categorized into two groups based on income: low-income and high-income. The results of this income-based heterogeneity analysis are reported in Table 8, column (1). The findings reveal that staple food consumption patterns significantly influence food waste, particularly among high-income households, with statistical significance at the 5% level. This pattern likely stems from the tendency of rice-based, high-income households to purchase larger food quantities and prepare a broader variety of dishes to satisfy dietary preferences, thereby elevating the risk of waste. Conversely, low-income households exhibit greater price sensitivity, prompting more budget-conscious purchasing decisions and efforts to fully utilize ingredients, thus reducing waste.

The secondary source of heterogeneity is the presence of children in the household, a factor previously linked to variations in food waste behavior. Households were divided into two groups: those with children and those without. The heterogeneity analysis is detailed in Table 8, column (2). The results indicate that rice-based households with children generate significantly more food waste than wheat-based households without children. This disparity can be attributed to the need to procure a wider range of foods to meet children’s diverse dietary preferences, often resulting in over-purchasing and subsequent waste. Additionally, picky eating habits among children frequently lead to uneaten portions, while challenges in accurately estimating children’s consumption needs contribute to over-preparation, further exacerbating food waste in these households.

## 5. Discussion

### 5.1. Food Waste Generation

This study identifies a significant relationship between regional staple food consumption patterns and food waste among rural households in northern and southern China, a disparity attributable to variations in dietary culture. Qian (2021) [36] observes that individuals in southern regions tend to waste more food on average than their northern counterparts. Consequently, this research establishes a clear linkage between regional dietary culture and food waste levels. Luo’s findings further indicate that rural Chinese households waste an average of 1.67% of their food daily, with southern households exceeding this benchmark.

Heterogeneity analysis reveals that the influence of staple food consumption patterns on food waste is particularly pronounced in high-income households and those with children. High-income households often prepare a wider variety of foods to accommodate diverse dietary preferences, frequently resulting in over-preparation and subsequent waste. This finding is consistent with the existing literature, which suggests that over-preparation, rather than matching cooking quantities with actual consumption, is a common practice (Pearson et al., 2017) [61]. Similarly, households with children exhibit higher waste levels than those without, as they prepare nutrient-rich meals to meet children’s needs, but struggle to accurately predict intake, leading to excess preparation and waste.

Furthermore, this study underscores the role of planned cooking in significantly reducing food waste. Thoughtful menu planning mitigates unnecessary spoilage, consistent with the theory of planned behavior (Coşkun et al., 2020; Visschers et al., 2016) [62,63]. Additionally, larger household sizes correlate strongly with elevated food waste, a finding corroborated by existing research (Attiq et al., 2021) [64]. In larger families, cultural practices—such as preparing an extensive array of dishes for events like birthday celebrations—can unintentionally amplify waste, despite underlying good intentions.

China, as a country with a geographical span of over 5000 km and 56 ethnic groups, exhibits significant regional characteristics that influence household staple food consumption patterns and food waste behaviors. In the northern regions, the annual consumption of wheat-based products is much higher than in the south, while rice consumption follows the opposite distribution. This structural difference leads to fundamentally different mechanisms driving food waste in various regions. For instance, families in the North China Plain are more likely to generate raw material waste in the initial processing stages due to the complex production methods for wheat-based foods, whereas families in the Yangtze River Basin experience higher levels of product waste due to the improper control of rice cooking quantities. Additionally, the generational behavior heterogeneity brought about by rapid urbanization warrants attention; younger families in the more developed eastern regions, with increased reliance on convenience foods, tend to waste more compared to traditional purchasing methods. The interplay of spatial heterogeneity and cultural inertia explains the higher standard deviation of food waste at the national level and provides empirical evidence for the design of differentiated policies in the future.

### 5.2. Potential Policy Implications

This study explores the impact of staple food consumption patterns on food waste, highlighting the need for coordinated efforts between rural households and governments to address this issue effectively. Both parties play crucial roles in tackling food waste, and their actions should be aligned. Several potential interventions for reducing food waste are proposed.

First, the government should facilitate greater mobility between the northern and southern regions to promote a broader understanding of dietary cultures. This can help reduce the dominance of traditional food cultures while encouraging diversity in food choices. Additionally, it is essential for policymakers to raise awareness about the harmful effects of food waste, particularly in the southern region, and to educate residents on the importance of food preservation.

Second, policymakers could implement cooking skills training programs for rural households involved in food production. These programs would aim to reduce the food waste caused by over-preparation by teaching households how to plan and prepare meals more efficiently.

### 5.3. Limitations and Suggestions for Future Studies

This study acknowledges several limitations that warrant consideration. First, it examines food waste differences among rural Chinese households from a cultural perspective, focusing specifically on the North–South dietary divide. However, the analysis is confined to the household level and excludes waste occurring in other consumption contexts, such as university cafeterias or supermarkets.

Second, the data rely on self-reports from household respondents, which are susceptible to biases such as recall inaccuracies and social desirability effects, potentially leading to underreported waste. Variations in respondents’ standards for estimating waste may further compromise data accuracy.

Third, data constraints precluded the inclusion of variables such as income distribution, urbanization levels, and local dietary traditions as controls. Additionally, the absence of more recent data limits the study’s ability to capture temporal shifts in food waste behaviors. To address this, future research should leverage updated longitudinal datasets to validate and extend these findings. This study commits to bridging this gap in subsequent analyses by incorporating current data and conducting further investigations to ensure the findings’ ongoing relevance.

Fourth, the survey data omit waste from vegetables and fruits, likely resulting in an underestimation of total household food waste. Given the perishability and short shelf life of these items, they are particularly prone to spoilage. Moreover, accurately gauging appropriate purchase quantities poses challenges, often contributing to elevated waste levels.

To enhance the robustness and comprehensiveness of food waste research, future studies should address several critical issues. First, employing more recent survey data is essential to confirm the current applicability of these results and reflect contemporary trends. Second, adopting objective quantification methods—rather than relying on self-reported estimates—would yield more precise and reliable data. Third, expanding the scope to include a wider array of food types, particularly perishable items like vegetables and fruits, would provide a more holistic understanding of household food waste and improve waste estimates across categories. This study explicitly recognizes the limitation of excluding perishable foods and discusses its potential impact, noting that their waste patterns may diverge from those of other food types—a distinction warranting greater attention in future research. Furthermore, subsequent investigations should examine waste behaviors across food categories in greater detail, with a particular focus on perishables, while accounting for factors such as seasonality and storage conditions.

Finally, although this study controlled for key variables like household income and education, it could not account for regional urbanization levels, food storage infrastructure, or price differentials due to data limitations. The existing literature suggests that urbanization indirectly shapes household waste behaviors by altering food supply chains. For example, suburban rural areas with greater access to processed foods may exhibit distinct waste patterns compared to core rice-producing regions. Similarly, inadequate storage facilities heighten spoilage risks, increasing waste, while higher regional food prices may encourage more judicious consumption practices to minimize losses, driven by cost considerations. Although these factors were not the primary focus here, they likely interact with staple food consumption patterns in complex ways, potentially moderating or amplifying their effects on waste. Future research incorporating these dimensions could offer a more comprehensive understanding of household food waste drivers and mitigate confounding influences from these variables.

## 6. Conclusions

This paper examines household food waste across 28 provinces in rural China, focusing on how staple food consumption patterns influence food waste. The findings reveal that rice-based households generate more food waste than wheat-based households, a trend observed across multiple food categories, including rice, soybean products, pork, beef, poultry, and aquatic products. The study also highlights factors such as the education level of the household head, modern cooking skills, and planned cooking habits, which significantly contribute to reducing food waste. Additionally, the research identifies a positive relationship between household size and food waste. These results are further validated through robustness tests.

Heterogeneity analysis shows that the impact of staple food consumption patterns on food waste is especially prominent among high-income households and those with children. This analysis underscores the influence of dietary culture as a key factor in household food waste, providing new empirical insights into the underlying causes of food waste in rural China. This study proposes intervention measures to address food waste in high-income households. First, we recommend launching meal planning campaigns through community or online platforms to educate high-income households on optimizing food purchasing and consumption behaviors, thereby reducing waste caused by over-buying. Second, we suggest establishing a reward system to encourage portion control in high-income households, such as providing incentives through points or coupons for reducing waste. Additionally, we propose using digital tools (such as mobile applications) for data monitoring and feedback, helping households to track food consumption and waste and offering real-time feedback to raise awareness about saving. These measures are highly actionable and tailored to the characteristics of high-income households, providing practical solutions to reduce food waste effectively.

## Figures and Tables

**Table 1:** Summary of archeological evidence for the origin of cultivation crops in China.

Region	Date Range	Site	Evidence for Crops	Reference
Henan, Shaanxi, Shangdong	5000 BC	Miaodigou	Wheat seed impressions on burned clay	Li (1984) [38]
	2500–1900 BC	Liangchengzhen	Carbonized wheat	Crawford et al. (2005) [39]
	2562–2208 BC	Zhaojiazhuang	Carbonized wheat	Jin et al. (2011) [40]
	2400–2000 BC	Zhaojialai	Carbonized wheat stems	Huang (1991) [41]
	2200 BC	Jiaochangpu	Carbonized wheat	Zhao (2004) [42]
Gansu	2700–2350 BC	Xishanping	Wheat phytoliths	Li et al. (2007) [43]
	2026–1959 BC	Ganggangwa	Wheat	Dodson et al. (2013) [44]
	2135–1895 BC	Huoshiliang	Carbonized wheat	Dodson et al. (2013) [44]
	3645–1538 BC	Donghuishan	Carbonized wheat	Flad et al. (2010) [45]
Xinjiang	3092–1531 BC	Gumugou	Wheat	Wang (1983) [46]
	2011–1427 BC	Xiaohe	Wheat	Kaogusuo (2003) [47]
	2006–1622 BC	Xintala	Wheat	Dodson et al. (2013) [44]
Tibet	1439–929 BC	Changguoguo	Wheat	Fu et al. (2000) [48]

Source: Betts et al. (2014) [49].

**Table 2:** The definition and descriptive statistics of variables.

Variable	Definition	Mean	St. Dev.	Median
Food waste	Food waste ratio for the meal (%)	2.700	1.619	0.618
Staple food consumption pattern	1 if household is rice-based eaters, 0 if household is wheat-based eaters	0.661	0.474	1
Age	Age of household head (years)	57.299	9.718	50
Education	The schooling years of household head (years)	7.616	3.186	8
Cooking tools	1 if modern cooking tools are used when preparing staple foods, 0 otherwise	0.691	0.462	1
Food preference	1 if cooking with stir-fry accessories, 0 otherwise	0.767	0.423	1
Cook gender	1 if meal cook is male, 0 otherwise	0.086	0.280	0
Cooking habits	1 if dish made for one meal, 0 otherwise	0.727	0.446	1
Dining out	1 if household dines out, 0 otherwise	0.500	0.182	1
Cooking fuel	1 if household uses electricity as fuel for cooking, 0 otherwise	0.216	0.412	0
Condiment	1 if condiments are often used in cooking, 0 otherwise	0.892	0.310	1
Throw food	1 if unprocessed food is thrown away, 0 otherwise	0.034	0.182	0
Spicy food	1 if frequently eat spicy food, 0 otherwise	0.410	0.492	0
Average BMI	Whole family weight (kg)/square of height (meter)	0.592	0.170	0.627
Household size	Number of people residing in household (person)	4.596	1.711	5
Children number	1 if household has at least one child in school, 0 otherwise	0.137	0.344	0
Household income	Total household income (log)	11.100	0.578	11.099
Observation	1632			

**Table 3:** Rural household food waste rate in each region (%).

Area	Flour	Rice	Potatoes	Soybean	Pork	Beef	Poultry	Aquatic	Egg	All
Total	2.54	3.60	2.81	2.09	1.47	0.75	1.71	2.09	1.33	2.70
Rice area	2.34	4.50	3.10	2.06	1.49	0.51	1.75	2.34	1.39	2.97
Wheat area	2.75	2.46	2.49	1.85	1.42	0.99	1.77	1.67	1.23	2.32

**Table 4:** The impact of staple food consumption patterns on household food waste: baseline result.

Variable	Food Waste Ratio (%)	
	Estimate	Std. Error
Staple food consumption pattern	0.440 ***	0.126
Age	0.014 **	0.007
Education	0.037 *	0.019
Cooking tools	−0.219	0.198
Food preference	0.203	0.144
Cook gender	−0.278 *	0.163
Cooking habits	−0.021	0.135
Dining out	0.001 **	0.001
Cooking fuel	−0.021	0.031
Condiment	−0.129	0.135
Throw food	0.863 **	0.325
Spicy food	−0.156	0.123
Average BMI	−0.700 **	0.239
Household size	−0.129 **	0.037
Children number	0.129 **	0.151
Household income	−0.067	0.098
Constant	2.050 *	1.185
R^2^	0.034	
Observation	1632	

Note: * *p* < 0.1, ** *p* < 0.05, and *** *p* < 0.01.

**Table 5:** The impact of staple food consumption patterns on household food waste: food items.

Area	Flour	Rice	Potatoes	Soy	Pork	Beef	Poultry	Aquatic	Egg
Total	0.185 *** (0.045)	0.219 *** (0.065)	0.108 *** (0.030)	−0.067 * (0.033)	0.009 (0.039)	0.016 (0.020)	0.093 *** (0.026)	0.020 (0.026)	0.139 (0.028)
Control variable	controlled	controlled	controlled	controlled	controlled	controlled	controlled	controlled	controlled
R^2^	0.100	0.062	0.138	0.126	0.064	0.068	0.097	0.082	0.120
Obs	1632	1632	1632	1632	1632	1632	1632	1632	1632

Note: * *p* < 0.1 and *** *p* < 0.01. The values appearing in parentheses are standard errors.

**Table 6:** The impact of staple food consumption patterns on household food waste: robustness test.

Matching Method	Estimate	Standard Error
Nearest neighbor matching (1:1)	0.984 ***	0.753
Kernel matching	0.602 ***	0.146
Radius matching	0.605 ***	0.146

Note: *** *p* < 0.01.

**Table 7:** The impact of staple food consumption patterns on household food waste: robustness test II.

Variable	Estimate	Std. Error
Staple food consumption pattern	0.800 *	0.024
Control variable	controlled	controlled
Constant	0.749	0.259
R^2^	0.086	
Obs	1632	

Note: * *p* < 0.1.

**Table 8:** The impact of staple food consumption patterns on household food waste: heterogeneity analysis.

Variable	(1)		(2)	
	High-Income	Low-Income	Without Children	With Children
Staple food consumption pattern	0.597 ** (0.196)	0.190 (0.189)	0.112 (0.355)	0.498 ** (0.144)
Control variable	controlled	controlled	controlled	controlled
Constant	0.132 (2.333)	4.778 (2.753)	1.376 (1.433)	6.957 * (3.541)
Pseudo R^2^	0.050	0.022	0.003	0.026
Obs	820	812	1361	271

Note: * *p* < 0.1 and ** *p* < 0.05. The values appearing in parentheses are standard errors.

## Data Availability

The data presented in this study are available on request from the corresponding author due to privacy.
